# Silver Nanoprism Enhanced Colorimetry for Precise Detection of Dissolved Oxygen

**DOI:** 10.3390/mi11040383

**Published:** 2020-04-04

**Authors:** Yunfeng Zuo, Longfei Chen, Xuejia Hu, Fang Wang, Yi Yang

**Affiliations:** 1 Key Laboratory of Artificial Micro- and Nano-Structures of Ministry of Education, School of Physics and Technology, Wuhan University, Wuhan 430072, China; zuoyf@whu.edu.cn (Y.Z.); coiyhm@whu.edu.cn (L.C.); huxuejiawuda@whu.edu.cn (X.H.); wangfang2200@whu.edu.cn (F.W.); 2 Shenzhen Research Institute of Wuhan University, Shenzhen 518057, China

**Keywords:** optofluidics, dissolved oxygen, silver nanoprisms, colorimetry

## Abstract

Dissolved oxygen (DO) content is an essential indicator for evaluating the quality of the water body and the main parameter for water quality monitoring. The development of high-precision DO detection methods is of great significance. This paper reports an integrated optofluidic device for the high precision measurement of dissolved oxygen based on the characteristics of silver nanoprisms. Metal nanoparticles, especially silver nanoprisms, are extremely sensitive to their surroundings. In glucose and glucose oxidase systems, dissolved oxygen will be transformed into H_2_O_2_, which affects the oxidation and erosion process of nanoprisms, then influences the optical properties of nanoparticles. By detecting the shift in the plasma resonance peak of the silver nanoparticles, the dissolved oxygen (DO) content can be determined accurately. Great reconfigurability is one of the most significant advantages of the optofluidic device. By simply adjusting the flow rate ratio between the silver nanoprisms flow and the water sample flow, real-time continuous adjustment of the detection ranges of DO from 0 to 16 mg/L can be realized dynamically. The detection limit of this device is as low as 0.11 µM (3.52 µg/L) for DO measurement. Thus, the present optofluidic system has a wide range of potential applications in fields of biomedical analyses and water sensing.

## 1. Introduction

Dissolved oxygen provides the necessary biochemical environment for the survival of aquatic organisms and is an indispensable material for aquatic life activities [1,2]. Dissolved oxygen monitoring plays an important role in aquatic ecosystem quality assessment, aquatic science experiments and aquatic resource exploration [3,4]. Besides, dissolved oxygen is also a key parameter in on-chip biochemical systems and chemical processing [5]. Generally, the three primary testing methods for the determination of dissolved oxygen in water are the iodometric method, the electrode polarography method (so-called Clark electrode method) and the fluorescence lifetime method. The iodometric method has a wide detection range and high detection accuracy [6]. However, the iodometric method has the defects of cumbersome operation, long operation time and high professional requirements for the operator. The electrode polarography method is simple and fast [7,8]. However, the presence of telluride, oil, carbonate and algae in the water sample may cause clogging or even damage to the gas permeable membrane. In the actual measurement process, it requires frequent maintenance. The fluorescence lifetime method is a simple and fast approach [9]. However, the system-building is complicated, and the price is relatively high. Therefore, an economical, efficient, accurate and simple measurement of dissolved oxygen is of great value for meeting the needs of production and scientific research. For this reason, it is of vital importance to develop an accurate detection system for dissolved oxygen monitoring. 

Metal nanoparticles in different shapes and sizes have various unique properties [10], which enable them to be applied in diverse fields such as biosensors and nanomedicine [11]. Compared with other kinds of metal nanoparticles, silver nanoparticles have a stronger plasmonic interaction with light [12]. Particularly, triangular silver nanoprisms exhibit more LSPR bands due to their anisotropic morphology than spherical or quasi-spherical silver nanoparticles [13]. The extraordinary properties of silver nanoprisms make it a powerful tool for biosensing and molecule detecting [10,14,15]. Optofluidics is a new interdisciplinary research field that focuses on the amalgamation of optics and microfluidics [16]. This new field provides lots of unique advantages for enhancing the performance and simplifying the design of micro-electromechanical systems [17]. Over the past decades, this new field has been developing rapidly, and it has been applied in many areas such as biosensor [18], water purification [19], optical devices [20,21] and water sensing [22]. Nowadays, the field of soft optical materials has received wide attention [23]. Optical devices based on frontier soft optical materials exhibit high biocompatibility and special qualities [24]. It is promising and expected to broaden the horizon of optofluidics in biomedical utility [25]. Optofluidics integrates multiple disciplines, which can not only realize on-chip sample pretreatment, but also can be used for the rapid detection of various biochemical indicators quickly and accurately [26].

The combination of silver nanoparticles and optofluidic systems can bring new merits. In one way, optofluidics provides a stable condition for mental nanoparticles synthesis [27]. In the other way, silver nanoparticles will strengthen the capability of optofluidic systems for biochemical sensing [28]. Therefore, optofluidics and nanotech are perfect candidates to innovate the measurement of dissolved oxygen. Herein, we designed an integrated optofluidic chip combining nanoparticle synthesis and spectrum detection to realize the precision measurement of dissolved oxygen. Rapid synthesis of silver nanoprisms was realized in a single chip. Owing to the novel physical properties of silver nanoprisms, minute amounts of dissolved oxygen concentration changes in water can be detected. Compared to traditional methods, this optofluidic DO detector possesses advantages such as higher measurement precision (detection limit less than 3.52 µg/L), less sample and reagents consumption (level of microliter), small size, low cost, parallel processing of samples, adjustable detection range (0–16 mg/L), and easy integration. This method can overcome the deficiencies of the traditional equipment in practical applications and has an important application prospect in the field of water environment monitoring.

## 2. Working Principle and Design 

**Figure 1:**
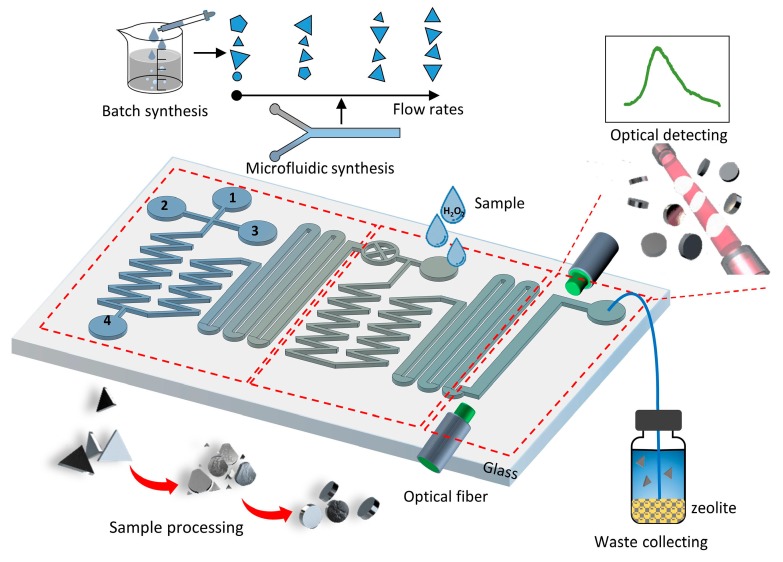
Schematic diagram of the optofluidic system based on Ag-nanoprisms etching for dissolved oxygen (DO) detection. This chip is consisted of three functional part: on-chip synthesis, sample processing, optical detecting. The detecting mechanism is based on the etching process of silver nanoprisms. The blue shift of the SPR peak of Ag nanoprisms can be used for the quantitative analysis of DO.

As illustrated in Figure 1, this integrated optofluidic system based on Ag-nanoprisms etching consists of three major parts: a silver nanoprisms synthesis module, a sample processing module and an optical detection module. The synthesis module was designed to realize the rapid, on-chip synthesis of silver nanoprisms. The geometric and optical features of silver nanoprisms can be controlled by adjusting the flow rate ratio of synthetic components rapidly and continuously. Through fast mixing in the microfluidic channel and precise control of reactant flows, flash chemistry can be achieved to realize rapid synthesis of nanoparticles in a single chip [29,30]. Compared with traditional batch synthesis methods, microfluidic methods process better synthesis efficiency [31]. The second part, samples mixing and processing module, is designed to realize fast and efficient mixing of pretreated water sample and synthesized Ag nanoprisms based on special microchannel design and fluid dynamics. Before being injected into the optofluidic chip, water samples with different DO contents were treated by a H_2_O_2_-generating system. In the presence of glucose (GO) and glucose oxidase (GOD), dissolved oxygen in the water sample is converted to H_2_O_2_ with concentration directly related to DO content. The reaction process is as follows:

(1) $$\beta \;glucose+O_{2}+H_{2}O\overset{GOD}{\to }\beta \;glucose\;acid+H_{2}O_{2}$$

where GOD is glucose oxidase that catalyzes the oxidation of β-glucose quickly and efficiently. The ratio of glucose and oxygen consumption to hydrogen peroxide product is 1:1:1. In this part, the pretreated water sample with generated hydrogen peroxide and Ag nanoprisms solution are injected into the system, and they are fully mixed and reacted in the channel. Micromixer allows reagents to be thoroughly mixed, which leads to controllable and stable reaction conditions. The Ag nanoprisms will be etched due to the presence of H_2_O_2_ in the pretreated water sample. The etching process is as follows:

(2) $$2Ag+H_{2}O_{2}\overset{\;}{\to }2Ag^{+}+2OH^{-}$$

Then, the mixture flows into the third part for optical detection. An optical detecting module combining the absorption cell is designed to capture the extinction spectrum of the eroded silver nanoparticles. The SPR (surface plasmon resonance) peak is extremely sensitive to the morphology of Ag nanoprisms. Different dissolved oxygen concentrations in the water will cause varying degrees of corrosion to the silver nanoparticles and affects the extinction spectrum, causing blue shifts of SPR peak. The detectable SPR shift signal can be used for the quantitative analysis of DO contents in water samples.

**Figure A1:**
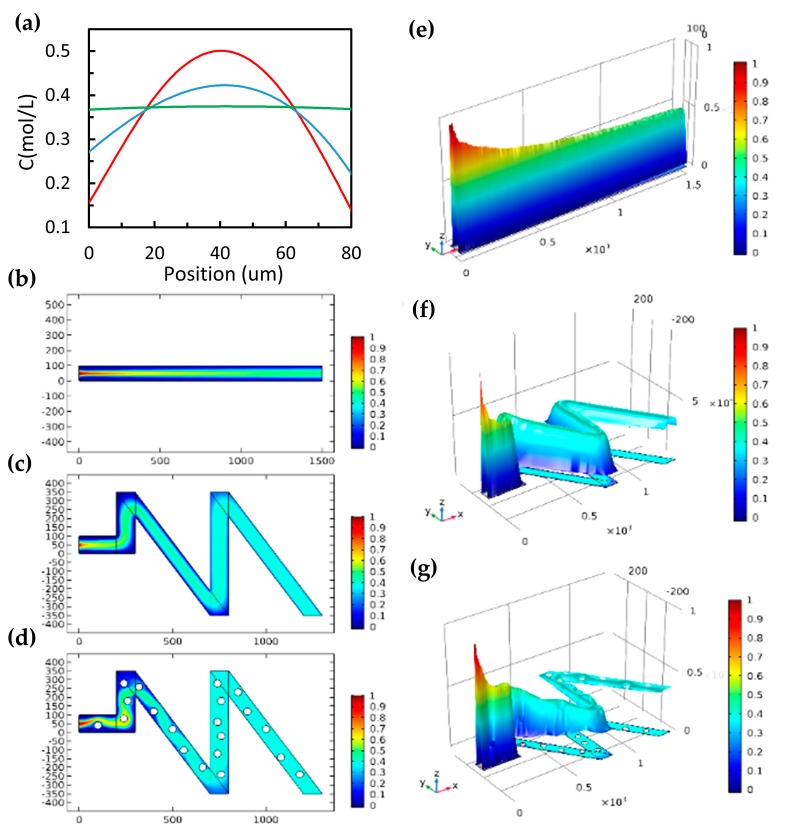
Simulation of liquids mixing in microchannel with different designs. (**a**) Cross-sectional concentration profiles of straight channel (red line), Z-shape channel (blue line) and Z-shape channel with blocks (green line) at x = 1000 µm. (**b**–**d**) Stable concentration distribution in main channel by simulation. (**e**–**g**) Corresponding height curves.

In this system, the full mixing of liquid reaction components or water samples and reagents is critical. Mixing efficiency affects not only the synthesis of nanoparticles, but also the accuracy of the system. Micromixers based on microfluidics are fundamental lab-on-a-chip components, which can realize the mixing of fluids within millisecond time scales to microsecond time scales, and have been applied in many fields [32]. Generally, micromixers can be divided into active and passive mixers. Active mixers rely on an external field, such as acoustic waves [33], to enhance the mixing and can achieve relatively high mixing efficiency [34]. Passive mixers realize fluid mixing based on structure design of the microchannel. Passive mixers are relatively simple and easy to integrate [35]. Among them, zigzag microchannel and microcylinders are usually applied to design passive micromixers based on chaotic fluid transformations [35,36]. Here, a passive micromixer based on a high-density Z-type (zigzag-type) hybrid structure was introduced, while microcylinders were designed in the Z-shape microchannel to accelerate the liquid mixing efficiency, ensuring that the reactants or samples were thoroughly mixed and reacted with each other quickly. Liquids mixing in microchannel with different designs were simulated by finite element method (FEM), as shown in Figure A1 (see Appendix A). According to the simulation, the Z-shaped channel with cylinders enables the high-speed mixing of liquids.

**Figure A2:**
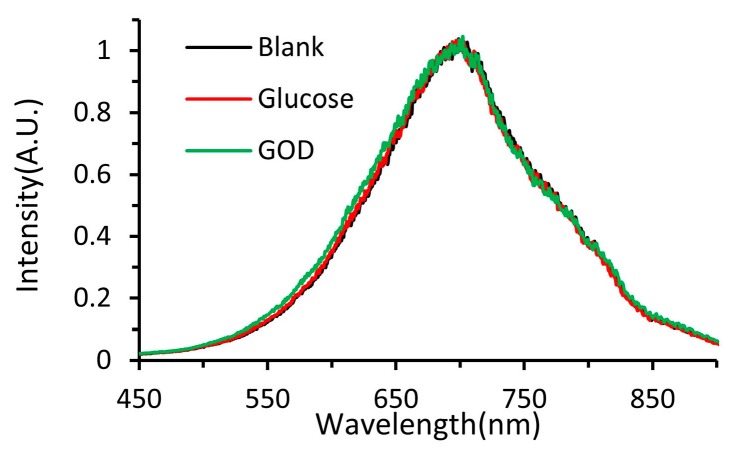
SPR extinction spectra of Ag nanoprisms in presence of glucose or GOD.

According to the chemical equation, the oxidization of glucose consumes O_2_ and generates H_2_O_2_. When the concentration of glucose is insufficient to consume all the O_2_ in sample water, the conversion degree of dissolved oxygen increases with glucose content. When the concentration is sufficient, the SPR peak shifts will remain the same. Under natural conditions, the concentration of DO is far less than 20 mg/L. To realize the precise detection of DO in water, a sufficient or excess amount of glucose is necessary; 20 mg O_2_ needs at least 0.625 mmol glucose. The content of glucose oxidase (GOD) is relevant to the reaction efficiency and reaction time. In consideration of the response time of the whole optofluidic system and the contents of DO in actual water samples, sufficient amounts of GOD solution (5 mg/mL) and glucose solution (2 mM) were added to the water sample with the ratio of 1:1:1. To investigate the stability of sliver nanoprisms and the assay system, the effects of detection reagents, glucose, GOD, were tested. As shown in Figure A2 (see Appendix A), no detectable SPR shift was observed, showing that GO and GOD formed a stable system with Ag nanprisms.

## 3. Experimental Results and Discussions 

### 3.1. Fabrication

**Figure A3:**
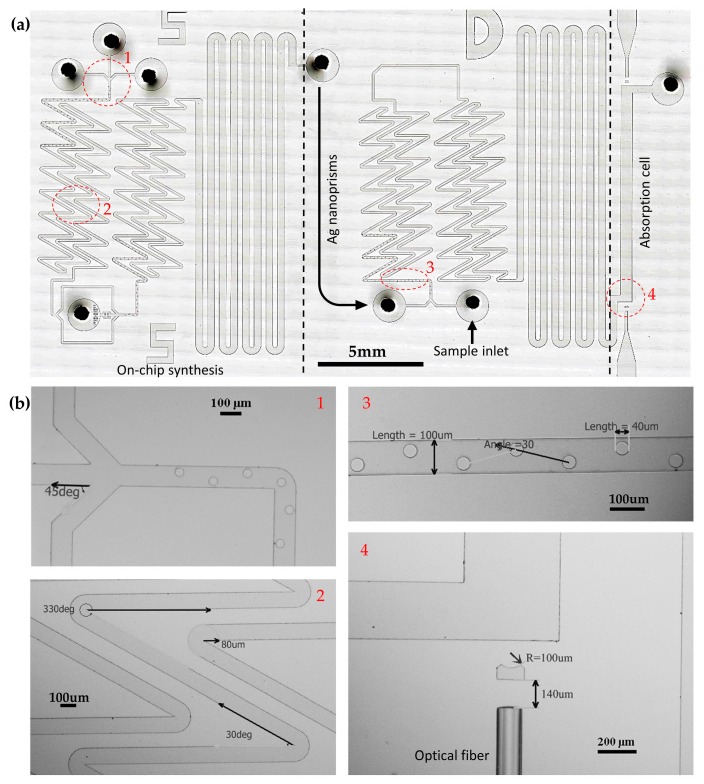
(**a**)Large Image of the fabricated chip obtained by image stitching technology and microscopes of the channel design at the positions of inlets (**b1**), zigzag-channel (**b2**), microcylinders (**b3**) and absorption cell (**b4**).

The optofluidic device was fabricated by polydimethylsiloxane (PDMS) using soft-lithography processes. First, a 50-µm layer of SU8 photoresist (SU8-2050, Microchem, Westborough, MA, USA) was spin-coated onto a silicon wafer. After pre-baking, the master was exposed to UV light under a glass mask using a mask aligner (H94-37, SVC, Chengdu, China). Then, through the process of post-exposure bake, development and hard bake, a thick, chemically and thermally stable image was constructed on the silicon wafer as a mold. Subsequently, the mold was covered with PDMS (Sylgard 184, Dow Corning, Midland, MI, USA) and heated at 75 °C for 1 h for polymer curing. Then the cured PDMS with microchannel pattern was removed from the silicon wafer and bonded onto a glass substrate after plasma oxidation (PDC-002, Harrick Plasma, Ithaca, NY, USA). Then, the fabricated PDMS chip was stored in an oven at 75 °C for 30min to increase the bonding strength. After inserting fluidic tubing and optical fibers into the microfluidic chip, the optofluidic chip was fabricated. Reagent flow streams were injected by using micropumps (LSP01-2A, LongerPump, Baoding, China). The width of the inlet and mixing channel was 100 µm, and the width of the main channel for the reaction was 300-µm. The height of the microchannel is 50 µm. A large image was obtained by inverted microscope (Ti-E, Nikon, Tokyo, Japan) and microscope image stitching technology (NIS-Elements AR, Nikon, Tokyo, Japan) to illustrate the fabricated optofluidic device, as shown in Figure A3a (see Appendix A). Figure A3b shows the microscopes of the channel design at the positions of the inlets, zigzag-channel, microcylinders and absorption cell.

### 3.2. Fast Mixing

**Figure 2:**
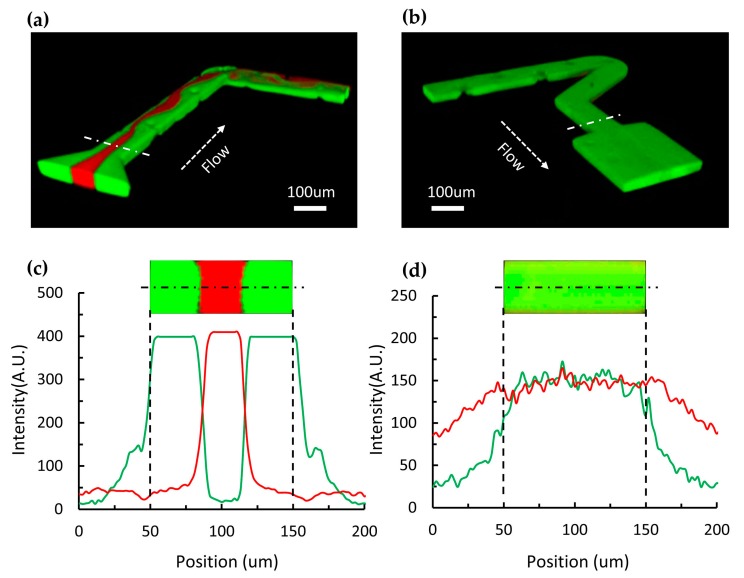
Mixing process of regent flows in microchannel. (**a**,**b**) Images of the three-dimensional liquids distributions capture by laser scanning confocal microscopy before and after the mixing process. The flow rates are fixed at 20 µL/min for each reagent inlet. (**c**,**d**) Cross-sectional liquids distribution corresponding to (**a**,**b**).

The performance of micromixers in the optofluidic systems is related to the control of Ag-nanoprisms synthesis and the reaction process between regents. To visualize the process of fast mixing in the microchannel, laser scanning confocal microscopy (A1R, Nikon, Tokyo, Japan) was used to capture the three-dimensional distribution of liquids in the microchannel. The core flow and sheath flows were dyed with Rhodamine B and Rhodamine 6G, respectively. The core flow emitted red fluorescence, and the sheath flows emitted green fluorescence. Figure 2a,b show the confocal images of liquids mixing process in the microchannel. The flow rates of the three liquid flows were 20 µL/min, respectively. The Z-shaped channel with cylinders induced the secondary flows in microchannel and increased the mixing efficiency [36]. Figure 2c,d show the intensity distribution profiles of the dotted lines in Figure 2a,b. It was obvious that the liquids were thoroughly mixed in the Z-shape channel. This microchannel design can realize the quick mixing of liquid reagents at a low flow rate, and the mixing time is about 40 ms. 

### 3.3. On-Chip Synthesis of Silver Nanoprisms

The silver nanoprisms were synthesized real-time and dynamically in the optofluidic chip according to a standard synthetic procedure at room temperature and in a neutral pH environment. [37]. Briefly, there are four fluid inlets in the synthesis module, three of which (i_1_, i_2_, i_3_) are at the front of the efficient micro-mixer. Another inlet (i_4_) was designed at the middle of the mixer. Silver nitrate (AgNO_3_, 0.4 mmol/L), sodium citrate (4 mmol/L), and H_2_O_2_ (0.6 wt%) were injected into the optofluidic device through inlet i_1_, i_2_, i_3_ with different flow rates (Qi_1_ = 20 µL/min, Qi_2_ = 30 µL/min, Qi_3_ = 10 µL/min), respectively. These three streams were fully and quickly mixed as soon as they flowed through the micro-mixer. Then sodium borohydride (NaBH_4_, 4 mmol/L) was injected through inlet i_4_ with flow rate at 20 µL/min. 

**Figure 3:**
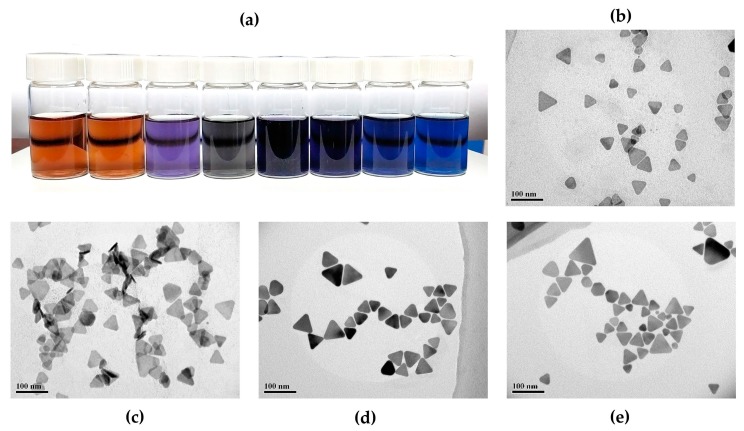
Synthesis of nanoprisms with different diameters. (**a**) The photograph of AgNPs synthesized through the lab-on-chip systems. TEM micrographs of the Ag nanoprisms under different synthesis conditions. Microfluidic synthesis with different flow rate ratios: Qi_1_:Qi_2_:Qi_3_:Qi_4_ = (**b**) 3:3:1:2, (**c**) 3:3:1.5:2, (**d**) 2:3:1:2. (**e**) Batch synthesis in macroscale.

**Figure A4:**
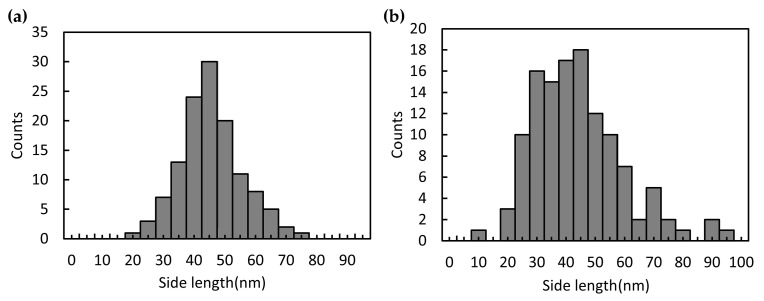
Particle size distribution of the Ag nanoprisms by means of microfluidics (**a**) and batch approach (**b**).

Through the synthesis module, on-chip synthesis of silver nanoprisms was realized, the geometric and optical features of silver nanoprisms can be controlled by adjusting the ratio of synthetic components rapidly and continuously. Silver nanoprisms with various diameters and SPR peaks can be synthesized through lab-on-chip systems, shown in Figure 3a. Figure 3b–e shows the TEM (Transmission Electron Microscope) micrographs of the Ag nanoprisms under different synthesis conditions. The TEM images were obtained using a JEM-2010 HT transmission electron microscope. Various shapes of silver nanoprisms were synthesized using the microfluidic method by simply changing the flow rate ratio between each regent (Qi_1_:Qi_2_:Qi_3_:Qi_4_ = 3:3:1:2, 3:3:1.5:2, 2:3:1:2), as shown in Figure 3b–d. Figure 3e shows the batch synthesis of silver nanoprisms in macroscale with the same reagents concentration as that in Figure 3d. The particle size distribution of the Ag nanoprisms synthesized by means of microfluidics and batch approach were studied, as shown in Figure A4 (see Appendix A). Owing to fast mixing in the microchannel and the precise control of reactant flows, the silver nanoprisms synthesized through the microfluidic method possess excellence reproducibility and narrow size distribution compared with batch synthesis method. The resultant Ag-nanoprisms solution was incubated for 24 h at room temperature before use, in order to eliminate the influence of borohydride, hydrogen peroxide and air bubbles. Then, the Ag nanoprisms solution was injected into the sample processing and optical detecting modules with an adjustable flow rate for DO measurement application.

### 3.4. Procedures for Dissolved Oxygen Sensing

To begin with, water samples with different dissolved oxygen concentrations were pretreated by injecting GOD solutions (5 mg/mL), glucose solution (2 mM), and oxygen-free water. The water sample with different DO contents and oxygen-free water were prepared through the aeration process by ultra-pure nitrogen and oxygen based on Henry’s law. Besides, all the reagents were prepared in a nitrogen glove box by using oxygen-free water and sealed hermetically before use to minimize the impact on detection accuracy. The pretreatment process was accomplished in a Z-shape microchip. The flow rate ratio between sample water, glucose, GOD and oxygen-free water was fixed at 1:1:1:7. Then, the mixed solutions were stored into an airtight glass syringe and incubated at room temperature for 20 min. According to the chemical reaction formula, DO was transformed into H_2_O_2_. Then, the post-treated water samples and Ag nanoprisms solution were injected into the optofluidic chip with different flow rate ratio (*r = Q_NPs_/Q_W_*_S_) according to the concentration of DO content. Under the condition of different flow rate ratio, the total flow rate was fixed at 60 µL/min. The reaction mixture solutions were mixed and reacted along with the microchannel. H_2_O_2_ etches the Ag-nanprisms to silver ions. Then the solution flowed through the absorption cell. A supercontinuum whitelaser (WhiteLase SC400, Fianium, Southampton, UK) was applied to be the light source for colorimetric, and the optical signal was received and transmitted by a multimode optical fiber. Then, the SPR spectra of the mixtures were measured by a spectrograph system including a CCD camera (Newton 920, Andor, Oxford, UK) with Andor’s line of Shamrock imaging spectrographs (Shamrock 303i, Andor, Oxford, UK). 

**Figure 4:**
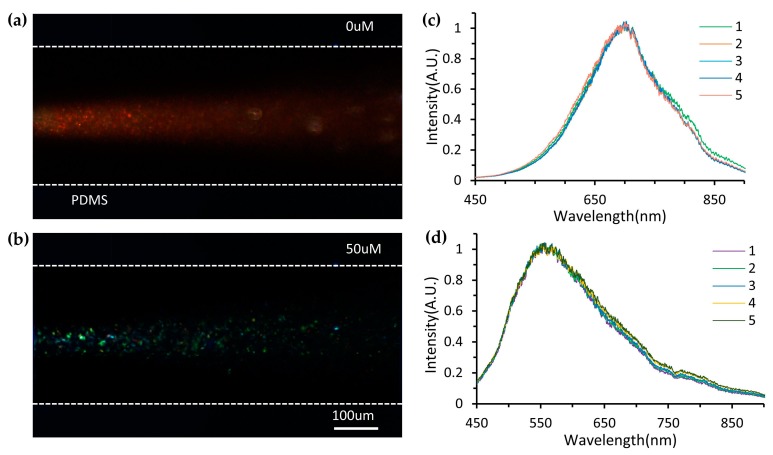
Images and spectra of the Ag nanoprisms illuminated by supercontinuum whitelaser. (**a**,**b**) Scattering images of silver nanoprisms before and after DO measurement (50 µM, 1.6 mg/L). And their corresponding normalized extinction spectra captured through the optical detection module (**c**,**d**). The spectra of the sample flow were collected five times, 10 s apart.

**Figure A5:**
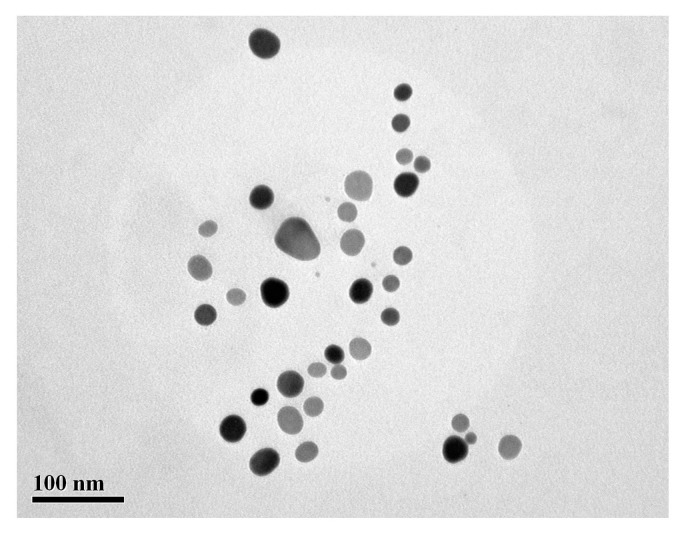
TEM micrographs of the Ag nanoprisms after the detection of dissolved oxygen (50 µM, 1.6 mg/L), the shape of Ag nanoprisms changed owing to the etching.

Figure 4 shows the images and spectra of the Ag nanoprisms illuminated by supercontinuum whitelaser. Scattering images of silver nanoprisms before and after DO measurement (50 µM, 1.6 mg/L) are shown in Figure 4a,b. Their corresponding normalized extinction spectra captured through the optical detection module are shown in Figure 4c,d. The flow rates of post-treated water samples and Ag nanoprisms solution were fixed at *Q_WS_* = 30 µL/min and *Q_NPs_* = 30 µL/min, respectively (*r* = 1). The spectra of the sample flow were collected 5 times, 10 s apart. By detecting the SPR peak shifts, the concentration of dissolved oxygen in the water will be determined. TEM was employed to characterize the morphological transition of Ag nanoprisms after DO sensing. The shape of Ag nanoprisms changed owing to the etching, as shown in Figure A5 (see Appendix A).

### 3.5. Device Performance

**Figure 5:**
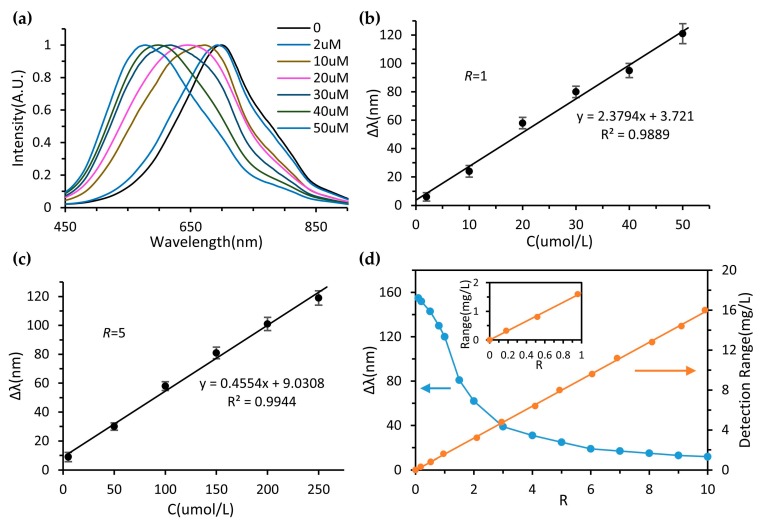
(**a**)Normalized SPR extinction spectra of the Ag nanoprism with different DO concentrations. Plots of SPR peak shift (Δλ) vs. concentrations of DO at different flow rates, (**b**) *r* == 1, (**c**) *r* = 5. (**d**) The blue line shows the relationship between SPR peak shift (Δλ) and flow rate ratio (*r)* when the concentrations of DO was fixed at 50 µM (1.6 mg/L). The orange line shows the detection range adjustment by changing *r*. The insert is an enlarged graph of the relationship between detection range and *r* (0–1).

In glucose and glucose oxidase systems, dissolved oxygen will be transformed into H_2_O_2_ and further affects the oxidation and erosion process of Ag nanoprisms. The precise and sensitive detection of dissolved oxygen is practicable based on the detection of blue shift in the plasma resonance peak of the Ag nanoprisms. The shifts of the SPR peak of silver nanoprisms were measured under different dissolved oxygen concentrations, and the integration time of the spectrometer was 20 ms, as shown in Figure 5a. The blue shift of the SPR peak increased with the concentration of DO. The flow rate ratio of Ag-nanoprisms solution and the post-treatment water sample (*r = Q_NPs_/Q_WS_*) was fixed at 1. The corresponding relationship between the SPR peak shift (Δλ) and concentration (C) of DO was shown in Figure 5b. A linear relationship was formed between the SPR peak shift and DO concentration ranging from 0 to 50 µM (1.6 mg/L). The linear relationship was expressed as Δλ = 2.3794C + 3.721 with a correlation coefficient R^2^ of 0.9889. By changing the flow rate ratio, *r = 5*, another linear calibration curve was acquired based on the SPR peak shift versus DO concentration, ranging from 0 to 250 µM (8 mg/L), as shown in Figure 5c. The linear relationship was expressed as Δλ = 0.4554C + 9.0308 with a correlation coefficient R^2^ of 0.9944. High reconfigurability is one of the most significant advantages of optofluidic systems. By simply adjusting the ratio (*r*) between silver nanoprisms flow and sample flow, the continuous adjustment of the detection ranges of DO from 0 to 16 mg/L can be realized dynamically, shown in Figure 5d. The blue line shows the relationship between SPR peak shift (Δλ) and flow rate ratio (*r)* when the concentration of DO was fixed at 50 µM. The orange line shows the detection range adjustment by changing *r*. The insert is an enlarged graph of the relationship between detection range and *r* (0–1). When detecting a water sample with the same DO concentration, Ag nanoprisms will be etched to a greater degree at a low flow rate ratio, which leads to a more obvious blue shift of SPR peak. A better detection limit can be achieved due to the greater response and sensitivity of the system at a low flow rate ratio. For the case of *r* = 0.5, a relatively low detection limit can be achieved. Under the established experimental conditions, a blank water sample was measured. After 10 measurements, taking the signal-to-noise ratio of 3, the system detection limit was calculated to be *D = 3σ/slope* = 0.11 µM (3.52 µg/L); *σ* is the standard deviation of the blank sample.

**Figure 6:**
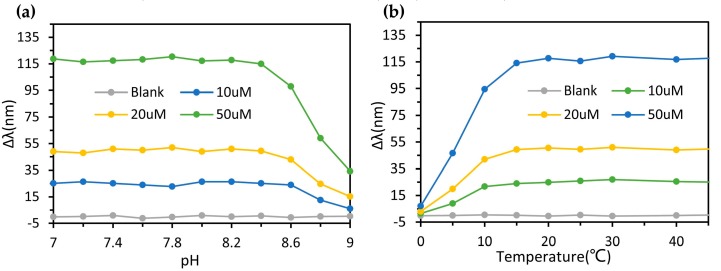
SPR peak shift of triangular silver nanoprisms under different conditions. (**a**) SPR peak shift of Ag nanoprisms versus pH value of sample water. (**b**) SPR peak shift of Ag nanoprisms versus incubation temperature.

The spectral characteristics of the silver nanoprisms under different pH values of sample water were measured, and the SPR peak shift as a function of pH was obtained. The effect of pH on the measurement results is shown in Figure 6a. Under different temperature conditions, the optofluidic device was also used to measure water with the same dissolved oxygen content. The change in the resonance peak of the silver nanoprisms was observed and recorded, as shown in Figure 6b. Under the circumstance of increasing pH value and decreasing temperatures, the SPR peak shift (Δλ) decreased. This was probably due to the impact on the activity of GOD under a high pH value and low-temperature condition. Besides, the detection system was not affected by pH value and temperature in a wide range, and exhibited relatively high stability. 

Since the ratio of nanoprisms and the water sample of the optofluidic device can be changed, a wide and adjustable detection range can be achieved. Compared with other iodometric method, electrode polarography method, and fluorescence method, the optofluidic method based on silver nanoprisms possesses better performance in dissolved oxygen detection Table 1 lists the performance of different DO detection techniques.

**Figure A6:**
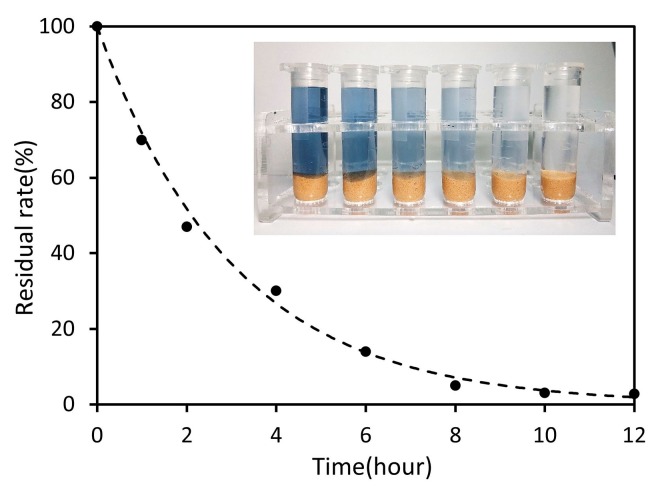
Absorption of the silver waste water by zeolite. Residual rate of silver nanoparticles as a function of time during the water purifying process.

In addition, the waste solution containing silver after DO measurement was collected and treated by zeolite [38]. The wastewater treatment process is shown in Figure A6 (see Appendix A). Then the absorbed silver was recycled by the precipitation method [39]. The rate of recovery can reach up to 98.3%, and the purity of silver powder can achieve 99.8%. The recovered silver can be recycled in DO measurement. This saves reagents, reduces detection cost and avoids environmental pollution.

## 4. Conclusions

In conclusion, we demonstrated a high-accuracy, eco-friendly optofluidic dissolved oxygen detector based on the etching of silver nanoprisms. The characteristics of the system and its anti-interference ability were investigated. For the measurement of dissolved oxygen, the detection limit is as low as 3.52 µg/L. The device possesses repeatability and optical stability. It is suitable for the measurement of dissolved oxygen under different environmental conditions. Compared with traditional dissolved oxygen determination methods, the optofluidic detector possesses a higher measurement resolution with a lower cost. Besides, the optofluidic device has advantages such as less reagent consumption and simple operation, and can realize precise detection and high adjustability in its detection range. High precision and reproducibility in the detection of DO is beneficial for researchers in helping them discover deep phenomena, conduct aquatic analysis, and reveal new laws in water environmental science. Therefore, the optofluidic dissolved oxygen detector has a good application prospect in water quality analysis.

Under the established conditions, the accurate detection of DO has been realized. In order to illustrate the effect of more factors such as the kinetics of mixing, total flow rate, micromixer design on the performance of the optofluidic DO sensor, further systematical investigation is necessary and is under conducting in our laboratory. Besides metal nanoparticles, there are still some materials, such as frontier soft optical materials, that are worthy of exploitation and have great potential in terms of biomedical sensing. This study will be beneficial to simplifying the system and optimizing the chip structure for various sensing applications. 

## Figures and Tables

**Table 1:** Comparison of optofludic method with other DO detection techniques.

Assay Methods	Sensitivity	Range	Detection Limit	Reference
Optofluidic DO detector	7.5 nm∙L∙mg^−1^	0–16 mg∙L^−1^	3.52 µg∙L^−1^	This work
Iodometric method	−	0-20 mg∙L^−1^	0.1 mg∙L^−1^	[6]
Electrode polarography	5.5 μA L∙mg^−1^	0.2-6.5 mg∙L^−1^	0.02 mg∙L^−1^	[8]
Fluorescence lifetime	I_0_/I = 117	0-40 mg∙L^−1^	−	[9]
